# A ruptured posterior communicating artery aneurysm presenting as tentorial and spinal isolated subdural hemorrhage: a case report and literature review

**DOI:** 10.1186/s12883-020-01682-8

**Published:** 2020-03-18

**Authors:** Abdulrahman Hamad Al-Abdulwahhab, Abdulaziz Mohammad Al-Sharydah, Sari Saleh Al-Suhibani, Abdullah Salman Almulhim, Obaied M. Al-Dhafeeri, Saeed A. Al-Jubran

**Affiliations:** 1 Diagnostic and Interventional Radiology Department, Imam Abdulrahman Bin Faisal University, King Fahd Hospital of the University, P.O. Box: 4398, Al-Khobar City, Eastern Province 31952 Saudi Arabia; 2 Diagnostic Imaging Radiology department, Royal Commission Health Services, Jubail, Saudi Arabia

**Keywords:** Clipping, Posterior fossa, Ruptured aneurysm, Saccular aneurysm, Subdural hemorrhage

## Abstract

**Background:**

Ruptured intracranial aneurysms are often associated with subarachnoid or intraparenchymal hemorrhage. However, the prevalence of subdural hemorrhage post aneurysmal rupture is low and rarely reported in scientific studies. Here, we report an unusual case of a ruptured posterior communicating artery aneurysm resulting in an isolated subdural hematoma located in the tentorial and spinal canal without subarachnoid or intraparenchymal hemorrhage.

**Case presentation:**

In this case, a 34-year-old woman with no history of trauma or coagulopathy was diagnosed with a subdural hematoma in the tentorium cerebellum tracing to the subdural space of the spinal column. Computed tomography angiography was used to identify the source of the bleeding, which revealed a ruptured left-sided posterior communicating artery saccular aneurysm. The aneurysm was clipped, and the hematoma was evacuated. The patient recovered without any neurological complications.

**Conclusions:**

Our results suggest that a diagnosis of ruptured intracranial aneurysm should be considered in patients with nontraumatic subdural hematoma. Prompt diagnostic imaging and interventional diagnostic procedures are required to ensure proper management of these patients and to avoid unnecessary complications.

## Background

Ruptured intracranial aneurysms are often associated with subarachnoid or intraparenchymal hemorrhage. The incidence of a ruptured intracranial aneurysm associated with an acute subdural hematoma is approximately 8%. However, the prevalence of an isolated subdural hematoma without intraparenchymal or subarachnoid hemorrhage, and trauma or coagulopathy is extremely low [1, 2].

Rapid analysis of the brain, using different imaging modalities (plain computed tomography [CT] and CT angiography [CTA]), helps detect intracranial hemorrhage and the underlying vascular anomaly, particularly in the absence of a traumatic event or coagulopathy. CT and CTA are the most commonly performed techniques for such rapid evaluations. However, magnetic resonance imaging (MRI) and magnetic resonance angiography (MRA) are the best available non-invasive diagnostic tools for the assessment of intracranial hemorrhage and its underlying etiologies. A higher sensitivity has been reported for MRI than for CT in diagnosing intracranial hemorrhage [3]. Other invasive diagnostic modalities, such as digital subtraction angiography (DSA), remain the gold standard for the assessment of the aneurysmal sac. Collectively, these invasive modalities have a superior role, as compared to the non-invasive modalities, in helping physicians determine an appropriate patient management strategy [4, 5].

To the best of our knowledge, the presentation of a nontraumatic aneurysmal subdural hemorrhage has not been established in the clinical setting. While a few patients complain of nonspecific symptoms, such as chronic headache, nausea, and confusion, most patients are diagnosed as being critical; therefore, rapid diagnostic tools and surgical interventions are required to lower the high prospective mortality and morbidity rates [1]. Here, we describe a rare case adhered to STROBE guidelines of tentorial and spinal subdural hemorrhage caused by a ruptured aneurysm in the posterior communicating artery.

## Case presentation

A 34-year-old woman started to experience unfocused mild back pain and chronic headache for 2 months. The severity of these symptoms had increased in the past 2 days, and they were not relieved with oral analgesic medications. The patient had no history of recent trauma or receiving anticoagulant or antiplatelet therapy. Patient’s physical examination revealed mild tenderness of the cervical and thoracolumbar spine. Further hematogical analyses, including white blood cell count, red blood cell count, platelet count, hematocrit, red blood cell volume, and hemoglobin concentration, revealed no obvious abnormalities. All vital signs and the results of the neurological examination were normal.

**Fig. 1:**
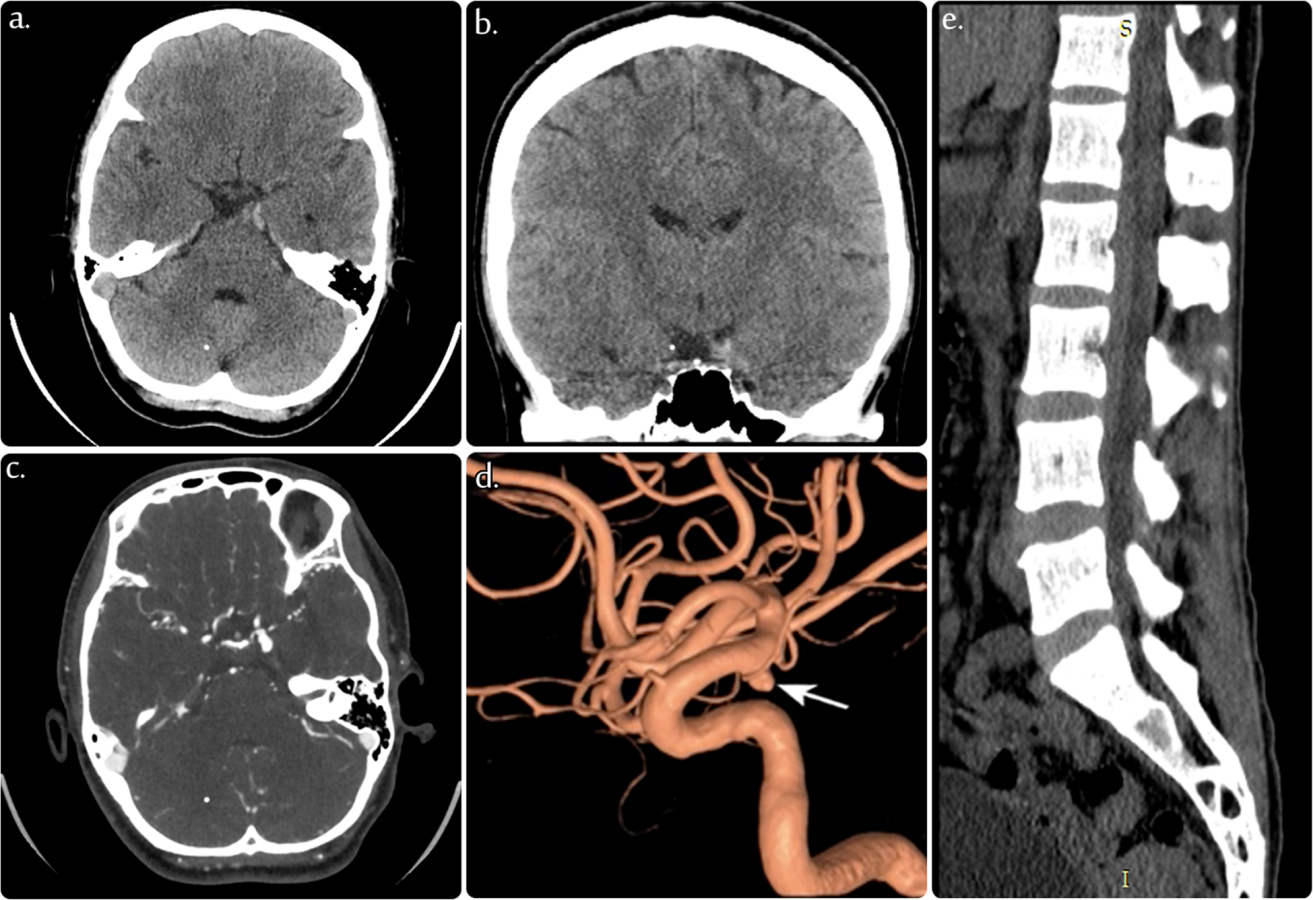
**a**, **b**, **c** Selective images of axial and coronal unenhanced computed tomography (CT) of the brain showing a small subdural hematoma at the anterior edge of the left tentorium, medial to the left uncus. **d** Selective 3D image of the left internal carotid artery (ICA) demonstrating unilocular posterior communicating artery (PCOM) aneurysm. (e) Selective image of the sagittal CT of the lumbar spine showing a subdural hematoma exerting a mass effect on the spinal canal

The initial unenhanced CT scan of the brain (Fig. 1a-b) revealed a small extra-axial hyperdense area located at the left anterior edge of the left tentorium cerebellum, medial to the left uncus, representing a small subdural hemorrhage. However, no subarachnoid or intraparenchymal hemorrhage was detected. CTA of the brain (Fig. 1c-d) revealed a wide-neck saccular aneurysm originating from the left posterior communicating artery. CT scans of the lumbar spine (Fig. 1e) showed a subdural hemorrhage, likely a continuum of the subdural hemorrhage of the brain.

**Fig. 2:**
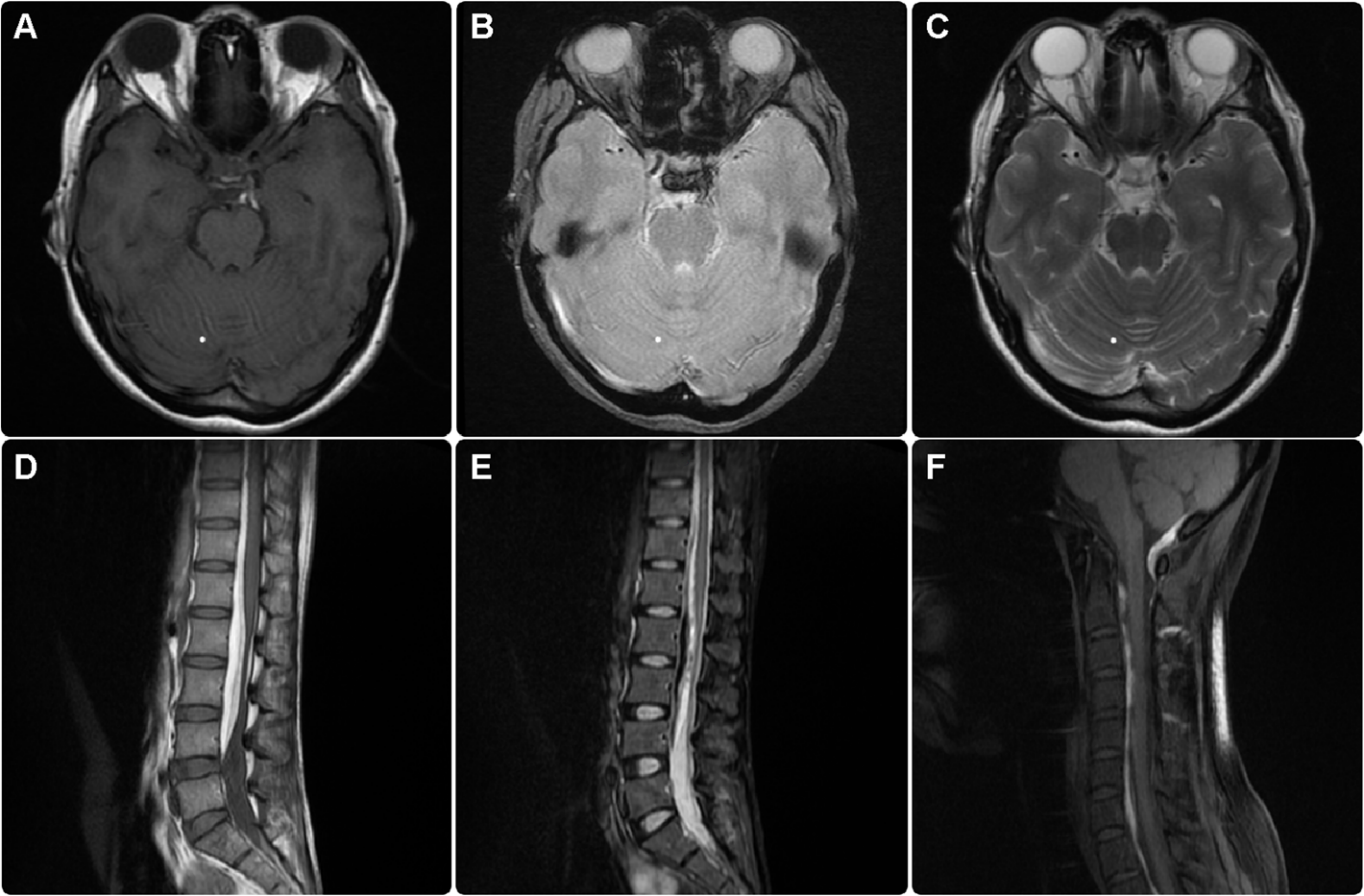
Multi-sequential magnetic resonance imaging (MRI) of the brain: axial T1-weighted (**a**), gradient (**b**), and T2-weighted (**c**) images showing a small area of extra-axial hyperintensity in the T1 image, and iso- to low-signal intensity in the T2 image, and a susceptibility artifact located at the anterior left tentorial edge, medial to the uncus, representing a subacute to chronic subdural hemorrhage in the gradient image. Multi-sequential MRI of the spine: sagittal T1-weighted (**d**), short tau inversion recovery (STAIR) of the lumbar spine (**e**), and T1-weighted fat saturation (**f**) images of the cervicothoracic spine showing massive extradural (subdural) bleeding presenting as predominantly high signal intensity on T1 (d) and predominantly low signal intensity on T2 STAIR (e) images, which show blood collection along the spinal canal extending from the posterior foramen magnum down to the lumbar spine, thus representing subdural hemorrhage

**Fig. 3:**
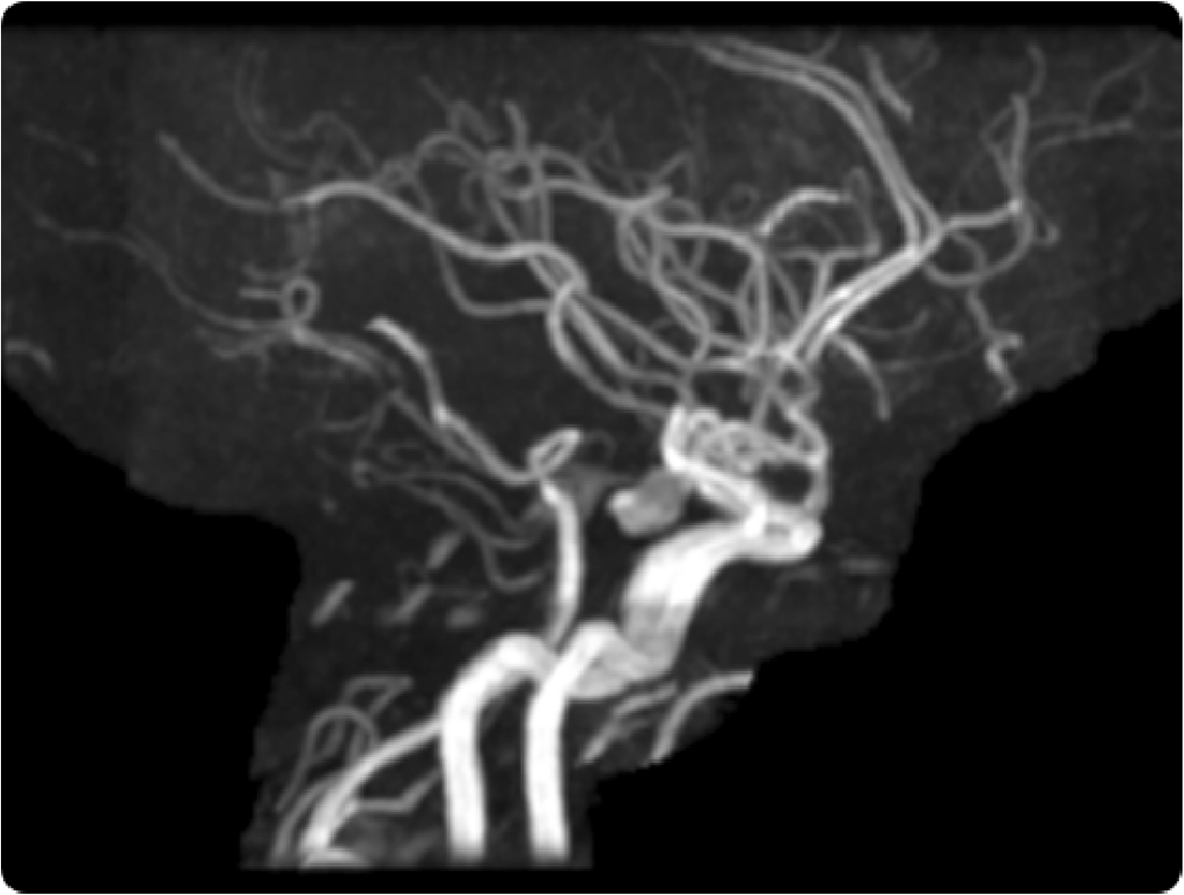
Magnetic resonance angiogram (time-of-fight) showing a large aneurysm in the left posterior communicating artery

Subsequent exploratory advanced imaging studies, including MRI of the brain and spine, revealed the presence of a thin layer of blood collection along the dual lining of the tentorium, down to the foramen magnum and extending up to the cervical and dorsolumbar spine. Mixed signal intensity on the T1- and T2-weighted images indicated late subacute or chronic hemorrhage (Fig. 2a-f). MRA of the brain revealed a large unilocular wide-neck posterior communicating artery aneurysm. There was no evidence of a separate aneurysm, vascular anomalies, brain edema, and established infarction (Fig. 3).

**Fig. 4:**
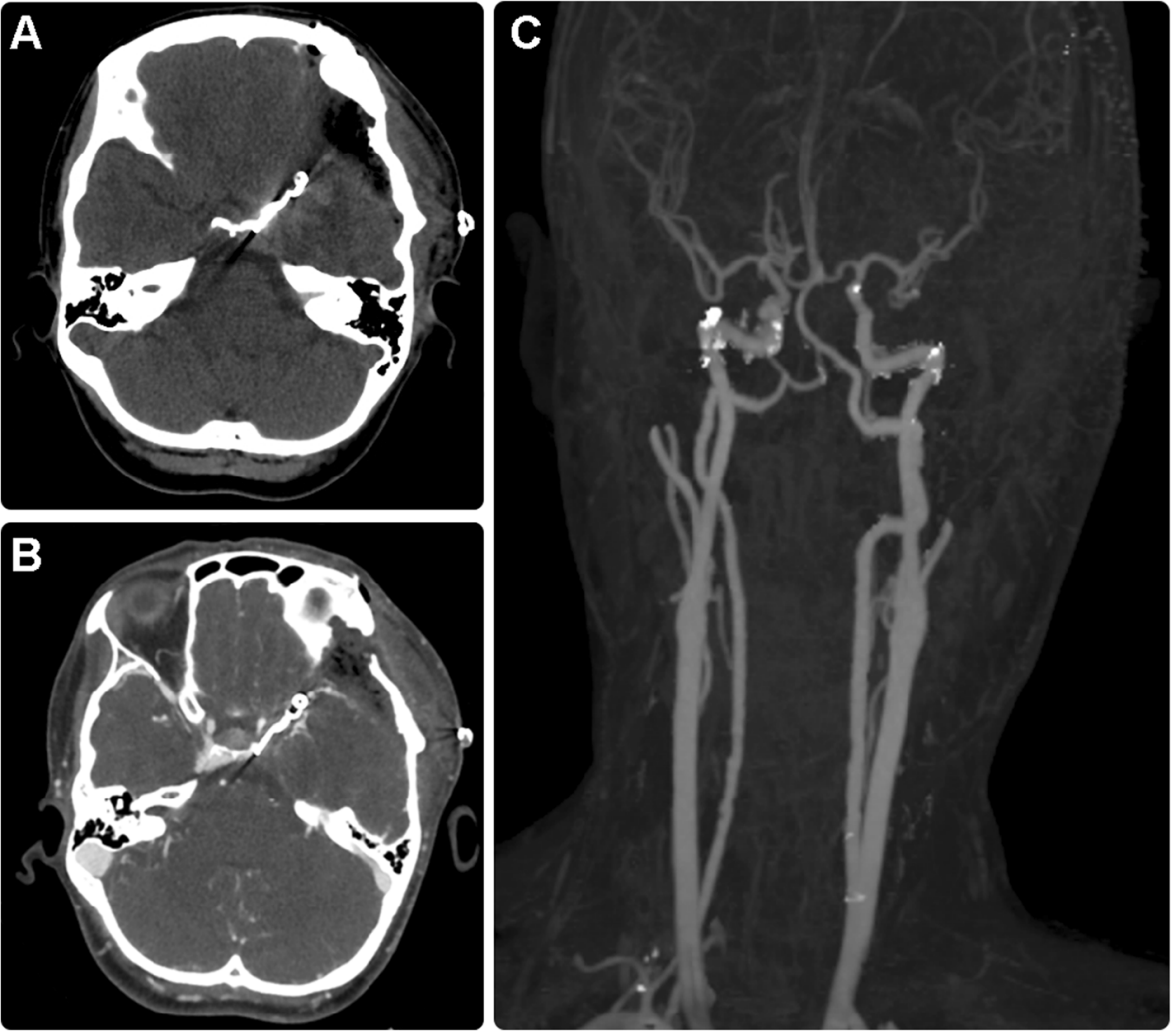
Axial unenhanced computed tomography (CT) scan of the brain (**a**), axial CT angiogram (**b**), and maximum intensity projection image (**c**) showing clipped matter in situ with no evidence of aneurysmal sac recanalization or recurrence. Left frontotemporal craniotomy is shown with the expected postoperative changes

An emergent clipping of the aneurysm and evacuation of the underlying cerebral hematoma were performed without any complications. The operating neurosurgeon confirmed that the rupture site was clotted and located in the lateral wall, close to the aneurysmal neck region. The patient completely recovered after the procedure. Immediate follow-up CT and CTA of the brain (Fig. 4a-c) revealed no evidence of a recanalized aneurysm sac or recurrence. No neurological complications or persistent symptoms were observed for 2 weeks post surgery, and consequently, the patient was discharged. A detailed and prolonged follow up (every month for the first 6 months and every 3 months thereafter) in the neurosurgery clinic for 2 years showed no further complications.

The patient was kept under clinical observation, as per the recommendation of the neurosurgeon, to monitor for any neurological complications that could have resulted from the subdural hematoma in the spine. To prevent reactive meningopathy, the patient received corticosteroid treatment to inhibit inflammation that could also play a key role in the hematoma formation [6].

The patient provided written informed consent for the publication of this case report.

## Discussion and conclusions

Subdural hematomas are commonly observed in elderly patients after brain trauma that disrupts the superficial bridging veins, commonly located along the cerebral convexity. However, it is rarely observed in cases of ruptured intracranial aneurysms. As reported in a previously published case, a subdural hematoma usually occurs because of a ruptured aneurysm in a major intracranial artery of the vertebrobasilar or carotid systems [5].

The pathogenesis of subdural hematoma development secondary to aneurysmal ruptures is not well established [7]. However, the commonly accepted mechanism involves a breach of the arachnoid membrane due to the rapid accumulation of blood under local stress conditions. This could then lead to adhesion of the arachnoid to the aneurysmal dome, thereby inducing a direct rupture of the aneurysm into the subdural space [8, 9]. In our patient, because of her aneurysmal configuration, we believe that the posterior communicating artery aneurysm expanded more into the posterior-lateral direction and was hindered by the petroclinoid band of the anterior tentorial edge (Fig. 1), with subsequent stretching and mechanical stress against the dura matter, thus resulting in the development of a fistula into the subdural space.

We think that our case is unique because it involves subdural hematoma in the absence of trauma or history of an altered coagulation state. Additionally, the severity of bleeding at the time of clinical manifestation ranged between subacute and chronic (Fig. 2). These observations are in line with those in previously published studies (Table 1) that reported only an acute phase hematoma, further emphasizing the uniqueness of our case. On the basis of previously published studies and our case, we conclude that the time interval from the rupture of an aneurysm to hematoma development is not directly correlated with the resultant mortality and morbidity.

**Table 1:** Cases of isolated subdural hematoma secondary to posterior communicating artery or posterior communicating artery-internal carotid artery aneurysmal rupture

No.	Author(s), year	Age, sex	Presentation	SDH location	Management	Prognosis
1	Araki et al. 2002 [10]	55/F	Headache, ptosis, and semicoma	Convexity	Clipping and hematoma evacuation	Good
2	Blake et al. 2003 [11]	35/F	Coma	Convexity	Nil	Dead
3	De Blasi et al. 2010 [4]	47/F	Headache and stupor	Convexity	Coil embolization	Good
		60/F	Headache and right sixth cranial nerve palsy	Bilateral convexity	Coil embolization	Good
4	Eggers et al. 1982 [12]	34/F	Headache	Convexity	Hematoma evacuation	Good
5	Friedman et al. 1983 [13]	55/F	Headache	Tentorium and interhemispheric	Clipping	Good
6	Inamasu et al. 2002 [14]	28/F	Coma	Convexity	Hematoma evacuation	Dead
7	Ishibashi et al. 1997 [15]	54/F	Headache	Tentorium and convexity	Clipping and hematoma evacuation	Good
8	Ishikawa et al. 2000 [16]	62/M	Headache and ptosis	Tentorium and interhemispheric	Clipping	Good
9	Kim et al. 2012 [8]	83/F	Headache, dizziness, and nausea	Clival hematoma migrated to the spine.	Stent-assisted coil embolization	Good
10	Kondziolka et al. 1988 [9]	43/M	Coma	Tentorial and convexity	Clipping and hematoma evacuation	Good
		38/F	Coma	Tentorial and convexity	Clipping and hematoma evacuation	Disabled
11	Mansour et al. 2014 [17]	51/M	Headache followed by loss of consciousness and right anisocoria	Bilateral convexity	Coil embolization	Good
12	Marbacher et al, 2010 [18]	39/M	Coma and bilateral dilated fixed pupils	Not mentioned	Clipping and hematoma evacuation	Disabled
		45/F	Coma and dilated fixed right pupil	Convexity	Clipping and hematoma evacuation	Disabled
		68/F	Right oculomotor paresis	Not mentioned	Clipping and hematoma evacuation	Good
13	Mrfka et al. 2013 [2]	40/F	Headache, nausea, and vomiting	Convexity	Hematoma evacuation and coiling	Good
14	Nonaka et al. 2000 [19]	52/F	Coma	Tentorium and convexity	Clipping and hematoma evacuation	Good
15	Onda et al. 1989 [20]	44/F	Semicoma	Convexity	Clipping and hematoma evacuation	Disabled
16	Paramasivam et al., 2012 [21]	43/F	Severe headache	Tentorium	Coil embolization	Good
17	Sasaki et al. 2018 [22]	71/F	Headache and drowsiness	Convexity	Clipping and hematoma evacuation	Disabled
18	Satoh et al. 1999 [23]	58/F	Semicoma	Convexity	Clipping and hematoma evacuation	Good
		25/F	Headache	Convexity	Clipping and hematoma evacuation	Good
		25/F	Coma	Convexity	Clipping and hematoma evacuation	Good
19	Thapa et al. 2018 [5]	55/F	Loss of speech and right-sided weakness	Convexity	Clipping and hematoma evacuation	Disabled
20	Williams et al. 1983 [24]	18/F	Coma	Convexity	Clipping and hematoma evacuation	Disabled
21	Our case, 2019	34/F	Headache	Tentorium and spine	Clipping and hematoma evacuation	Good

*No*. Number, *SDH* Subdural hemorrhage, *F* Female, *M* Male

Moreover, our observations suggest that a subdural hematoma arising from an aneurysmal rupture in the posterior communicating artery that results in hemorrhage within the clival tentorial or spinal column has a relatively better outcome than a hematoma resulting in hemorrhage within the cerebral convexity or other parts originating from the anterior circulation such as the middle or anterior cerebral artery. Since the subdural layer of the spinal canal is in close communication with the posterior fossa, a less pronounced overall pressure effect of a contained subdural hematoma is observed on the adjacent tissues compared to that of a hematoma arising in the middle or anterior cerebral arteries that does not freely communicate with the spinal canal. In our patient, we observed a clotted rupture site in the lateral wall, near the aneurysm neck. This observation confirms the aforementioned mechanical theory regarding the pathogenesis of this rare type of subdural hematoma secondary to aneurysmal ruptures.

We suggest that the best management option should be selected on the basis of the conventional assessment of the aneurysmal shape, neck dimensions, dome-to-neck ratio, aspect ratio, amount of hematoma, and the overall physical condition in patients with neurologically stable symptoms. Additionally, clinicians should decide on the suitability of performing endovascular embolization following these assessments. In our case, we found that the aneurysm was amenable to endovascular treatment but the patient required evacuation of the excessive amount of subdural hematoma. Based on the patient’s age and preference and in view of recent evidence that supports microsurgical clipping over endovascular treatment in young patients [25], we performed a single-stage procedure with clipping and evacuation of the cerebral hematoma (Fig. 4).

However, the management strategy should be selected based on the evaluation of the subdural hematoma size, brain parenchymal integrity, and the hematoma mass in patients with unstable neurological symptoms. Craniotomy should be performed at the earliest to evacuate the hematoma initially, and subsequently, endovascular treatment can be performed, which remains the optimal option, if applicable [18, 26].

Many questions must be answered to better comprehend the pathological nature of such rare conditions. It is imperative to determine whether advanced imaging techniques can easily differentiate between subdural enhancement of the venous plexus and trace amounts of subdural hemorrhage in a common site like the retro-clival area. It is also equally pertinent to understand if other vascular lesions, such as an arteriovenous malformation, cavernoma, and arteriovenous fistula, which are located in the intra-axial location, can cause isolated subdural hemorrhage without intraparenchymal or subarachnoid hemorrhage.

In order to further our understanding of this rare disease condition, specialist neurological centers could recruit patients with similar symptoms to participate in prospective cohort studies and investigate long term issues and best management choices.

In conclusion, the prevalence of a subdural hematoma secondary to a ruptured intracranial aneurysm without intraparenchymal or subarachnoid hemorrhage is low. Most patients in the previously reported cases presented to the clinic late, with such cases often being associated with a poor neurological condition. The time of presentation is likely an independent predictor of patient outcome. Prompt investigation using advanced diagnostic imaging, while considering all possible differential diagnoses, is warranted to determine whether coiling angiography or aneurysmal clipping is a preferred treatment option. Finally, CTA must be performed in patients with a subdural hemorrhage in the absence of a traumatic event or coagulopathy. Further studies, involving a large patient cohort with similar symptoms, are required to understand long-terms implications and improve patients’ management.

## Data Availability

The datasets during and/or analyzed during the current study are available from the corresponding author on reasonable request.

## Abbreviations

CT: Computed tomography
CTA: CT angiography
DSA: Digital subtraction angiography
MRA: Magnetic resonance angiography
MRI: Magnetic resonance imaging
